# Supplementary material for: Actin monomers influence the interaction between Xenopus cyclase-associated protein 1 and actin filaments
Source: bioRxiv. 2025 Aug 15:2025.08.14.670363. Preprint. [Version 1] doi: 10.1101/2025.08.14.670363 (PMC12363965; doi:10.1101/2025.08.14.670363)
Supplement: Supplement 1 — Supplementary Figure 1. Classification of different molecular states of the XCAP-actin complex. Representative HS-AFM images (A), volume distributions (B), and models (C) of six different states of the XCAP1-actin complex on mica surfaces. Bar, 20 nm. Original scanning area was 150 × 150 nm2 with 80 × 80 pixels, and cropped size was 60 × 60 nm2. Imaging rate was 0.2 s/frame (5 fps). The images show that they are different only in the lateral arm domains representing the CARP domain of XCAP1 that reversibly interacts with G-actin (ref. 51). [file media-1.zip › Supplementary Fig1.pdf]

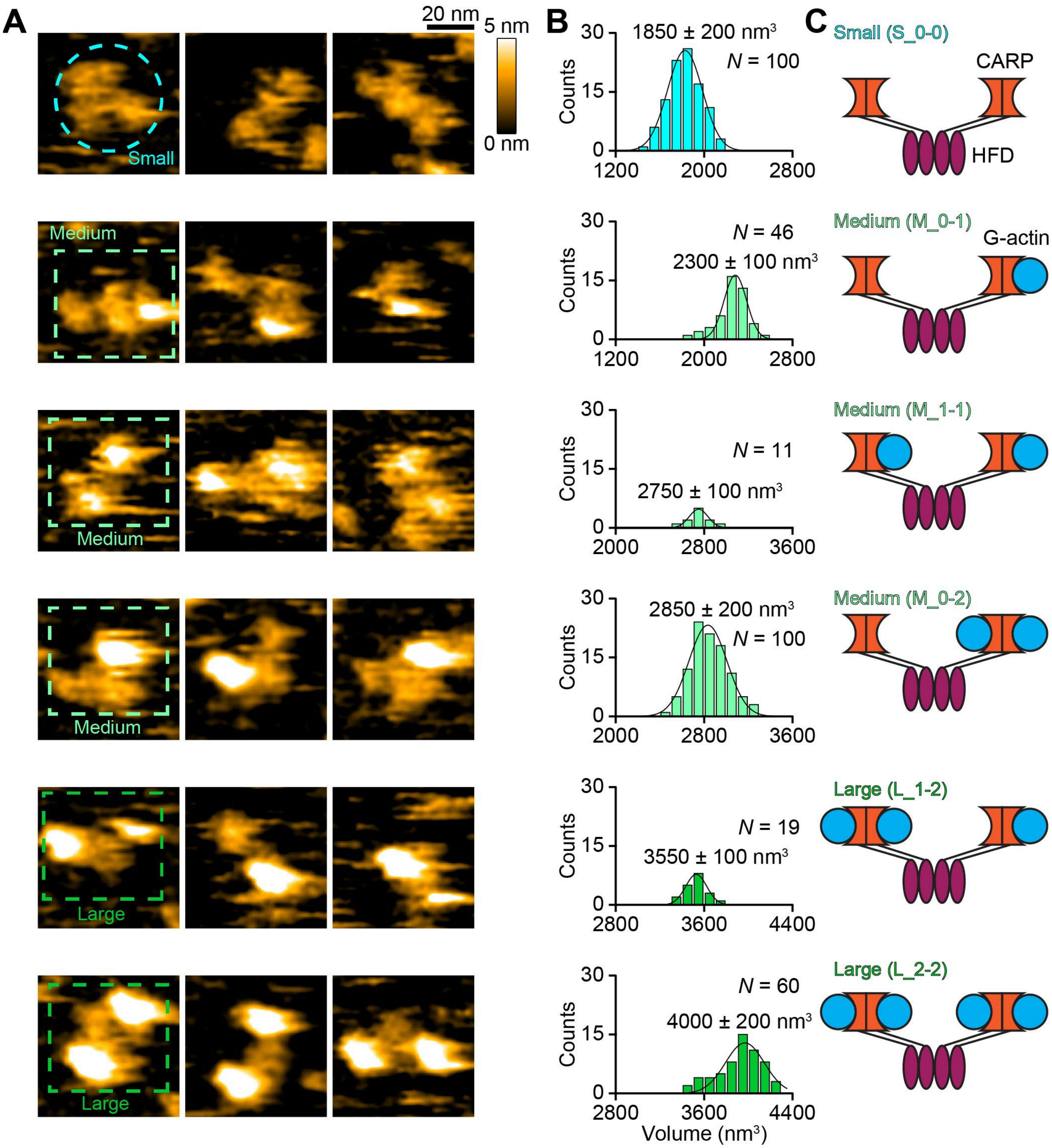

Supplementary Figure 1. Classification of different molecular states of the XCAP-actin complex. Representative HS-AFM images (A), volume distributions (B), and models (C) of six different states of the XCAP1-actin complex on mica surfaces. Bar, 20 nm. Original scanning area was 150 × 150 nm^2 with 80 × 80 pixels, and cropped size was 60 × 60 nm^2. Imaging rate was 0.2 s/frame (5 fps). The images show that they are different only in the lateral arm domains representing the CARP domain of XCAP1 that reversibly interacts with G-actin (ref. 51).
